# A Novel Technique for Retention of the Immediate Obturator Following Maxillectomy in Mucormycosis: A Case Report

**DOI:** 10.7759/cureus.24687

**Published:** 2022-05-03

**Authors:** Jyoti Paliwal, Vineet Sharma, Balwant Gurjar, Ramawatar Nagar

**Affiliations:** 1 Prosthodontics, Rajasthan University of Health Sciences (RUHS) College of Dental Sciences, Jaipur, IND

**Keywords:** covid-19, retention, magnets, immediate obturator, mucormycosis

## Abstract

Mucormycosis is an aggressive opportunistic fungal infection that affects blood supply-rich areas such as the maxilla. Because of the compromised immune system caused by coronavirus disease 2019 (COVID-19) infection and diabetes, this infection has spread at a rapid rate. Early detection and treatment can reduce disease mortality and morbidity. However, the difficulties of prosthetic rehabilitation and the lack of multidisciplinary planning negatively influence the quality of life (QOL). This case report uses the novel concept of magnet-retained immediate prosthetic rehabilitation in such a case.

## Introduction

Mucormycosis is an aggressive opportunistic fungal infection caused by the order Mucorales of zygomycete fungi. The fungus often attacks the sinuses and lungs due to their affinity for blood vessels. The primary site of infection is the maxillary antrum, with erosion, perforation, and necrosis of the affected tissue [1,2].

Following the second wave of coronavirus disease 2019 (COVID-19), many Indian states declared mucormycosis an epidemic [3]. The estimated prevalence of mucormycosis is 0.02-9.5 per 100,000 [4]. Diabetes, a weakened immune system, poor oral hygiene, a prolonged ICU/hospital stay, comorbidities, post-transplant, cancer, voriconazole therapy, renal failure, and high-iron overload may all be risk factors for post-COVID mucormycosis [1,2].

Mucormycosis affects the sinuses and lungs because of its affinity for blood vessel-rich areas [1]. The maxillary antrum is the standard site for the beginning of contamination [1,5]. Medical intervention with surgical debridement and resection surgery is used to treat rhino-orbital mucormycosis, which causes difficulty with speech, deglutition, and mastication [1,5]. This case report described the immediate surgical rehabilitation post-mucormycosis maxillary defect by using a magnet-retained immediate obturator.

## Case presentation

A 45-year-old male patient presented with pain and an ulcer in the upper anterior labial region and recurring right nasal discharge after meals. The patient was diagnosed as positive for COVID-19 one month ago and was treated for the same as per clinical guidelines. Following recovery, the patient complained of pain in the right temporal region and visited a physician. Laboratory tests revealed elevated glycosylated hemoglobin (HbA1c=10.7), blood sugar levels (149 and 269.39 mg/dl), and an elevated erythrocyte sedimentation rate (ESR) (58 mm/1st hour). CT scans revealed bilateral ethmoidal and maxillary sinusitis. The patient was referred to the prosthodontics department for prosthetic rehabilitation.

**Figure 1:**
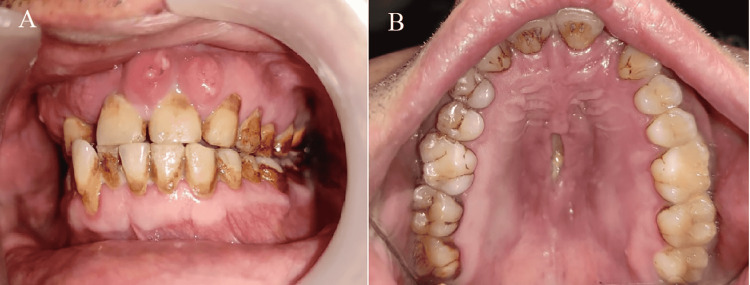
Intraoral view (A) poor oral hygiene and multiple sinus opening (B) blackish discoloration with denuded bone

On extraoral examination, the patient had diffuse swelling over the malar region of the face. Intraoral examinations showed a single 1.5 cm × 0.5 cm mid-palate ulcer with irregular necrotic borders. Although the palatal bone was exposed, the ulcer was non-tender. Multiple pus-draining sinuses were present in anterior teeth (13, 14, 21, 22, and 23) with grade 2 mobility of maxillary teeth (Figure 1).

**Figure 2:**
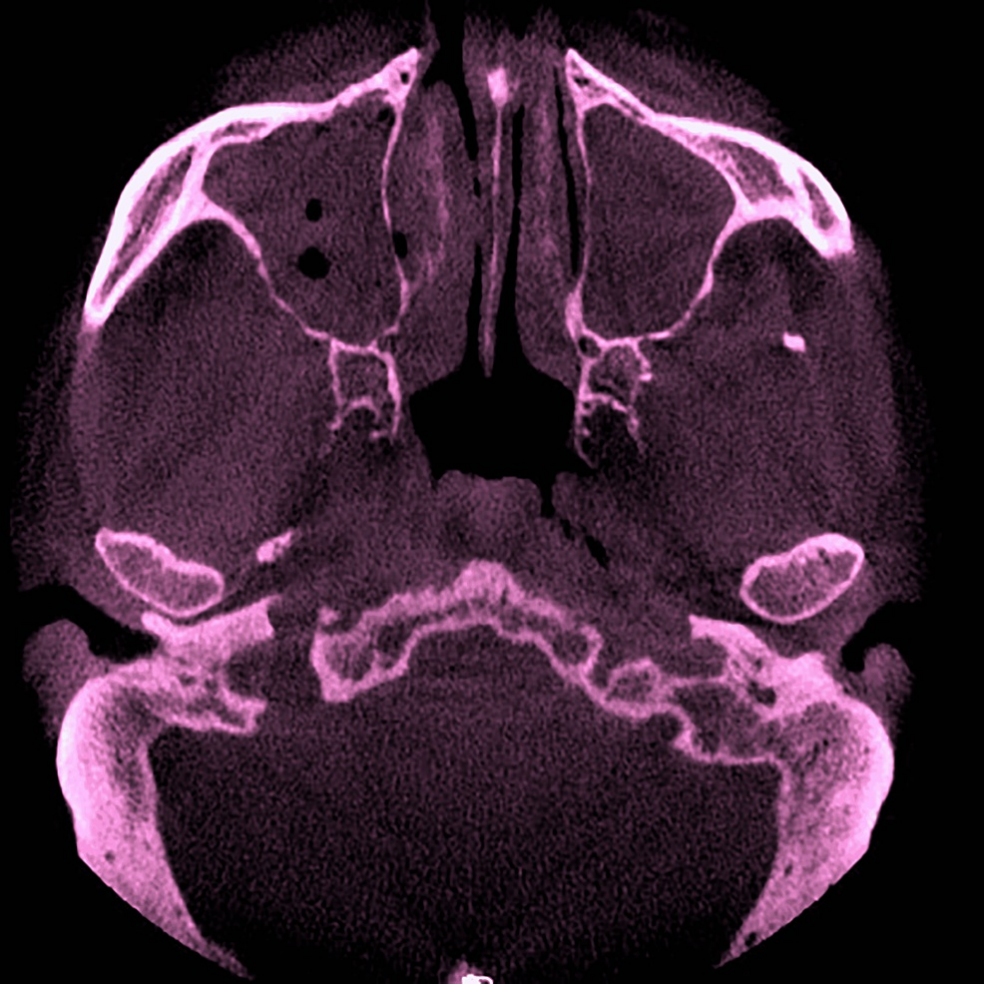
CBCT showing right and left maxillary sinus radio-opacity CBCT: cone-beam computed tomography

A differential diagnosis of (a) mucormycosis, (b) osteomyelitis, and (c) aspergillosis was made. Magnetic resonance imaging (MRI) of the paranasal sinus contrast revealed a heterogeneously enhanced large mixed-signal intensity soft-tissue lesion with infiltration of the lesion into the right masticator space and right maxilla, as well as evidence of peripherally enhanced mucosal thickening with fluid collection in the left maxillary, ethmoid, and bilateral sphenoid sinuses, representing sinusitis with an underlying infective etiology, probably invasive fungal infection. Cone-beam computed tomography (CBCT) revealed moth-eaten palatal and alveolar bone infecting the maxillary sinus, ethmoid sinus, and nasal turbinate (Figure 2).

A provisional diagnosis of mucormycosis was made, and a multidisciplinary approach was used to improve patient outcomes, including surgical resection, Le Fort I osteotomy, and prosthetic modification. The prosthetic modification included placement of reconstruction plates bilaterally on the zygomatic bone to aid in placing neodymium magnets, recontouring of reconstruction plates to anatomic contours, and stabilizing neodymium magnets on reconstruction plates for retention.

Clinical procedure

**Figure 3:**
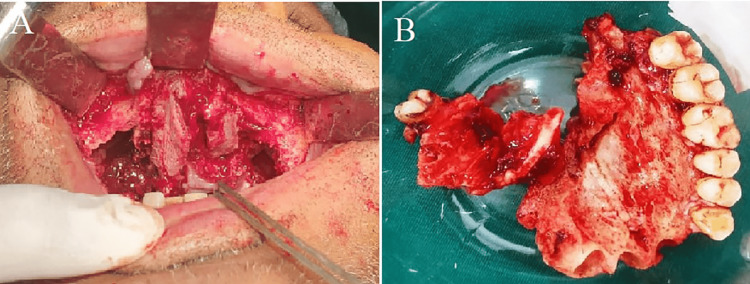
(A) Intraoral view: Le-Fort I osteotomy (B) maxillary segment: resected

**Figure 4:**
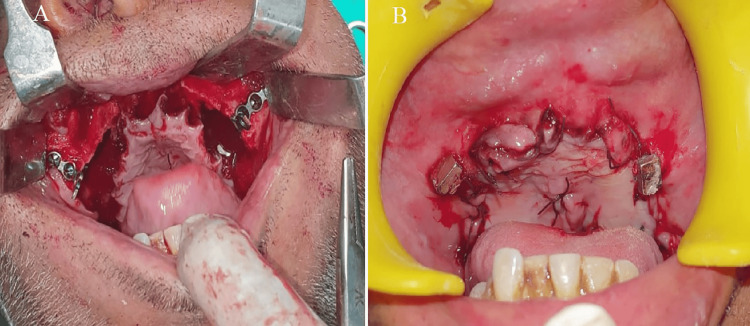
(A) Titanium reconstruction plate stabilized at the zygoma (B) neodymium magnets stabilized on reconstruction plates and primary closure done

The patient was prepared for surgery under local anesthesia. Le Fort-1 osteotomy (Figure 3) and surgical debridement were performed until the margins ensured healthy bone. Five and four-hole titanium continuous reconstruction plates were placed on the zygomatic bone. The plate was contoured within anatomical and physiological limits. Prefabricated neodymium magnets were stabilized using auto-polymerizing acrylic resin (Coltocure, Coltene India Ltd., Mumbai, India) mechanically with holes in the reconstruction plate with rubber-dam isolation to prevent a soft-tissue reaction. The magnets used were the 3 mm × 3 mm × 1 mm square-shaped neodymium iron boron (Nd-Fe-B) magnets (28AH-35AH Grade) with a maximum operating temperature of 230°C. Each magnet had a 1000-g attraction force. It employs a stainless-steel casing that is hermetically sealed by micro-laser welding to ensure excellent corrosion resistance. Primary closure was done (Figure 4). Counter magnets were oriented on the previously stabilized magnet on reconstruction plates as per the magnetic axis, and an irreversible hydrocolloid (Zelgan 2002, Dentsply, Delhi, India) pick-up impression was made.

**Figure 5:**
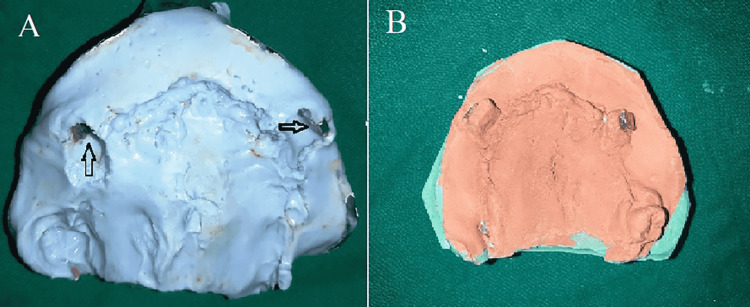
(A) Neodymium magnets for spatial orientation in irreversible hydrocolloid pick-up impression (B) neodymium magnets on working models

**Figure 6:**
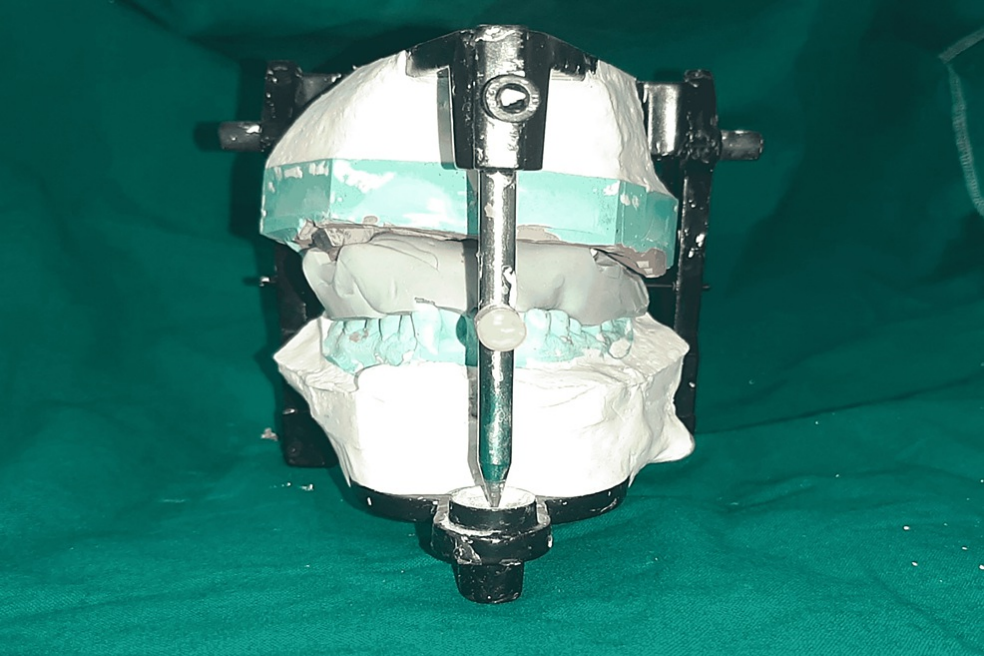
Mounted maxillary and mandibular casts using putty index

**Figure 7:**
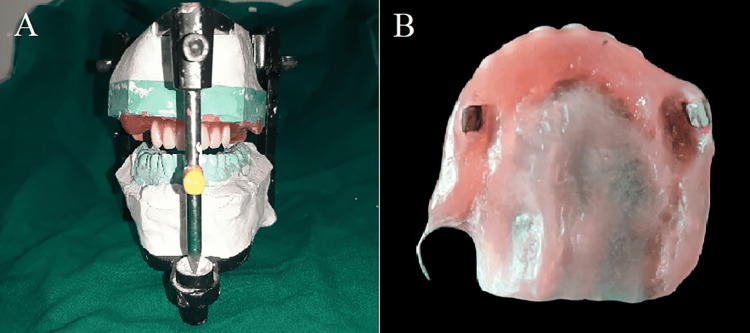
(A) Wax up of obturator with anterior teeth (out of occlusion) (B) intaglio of the obturator with processed neodymium magnets

**Figure 8:**
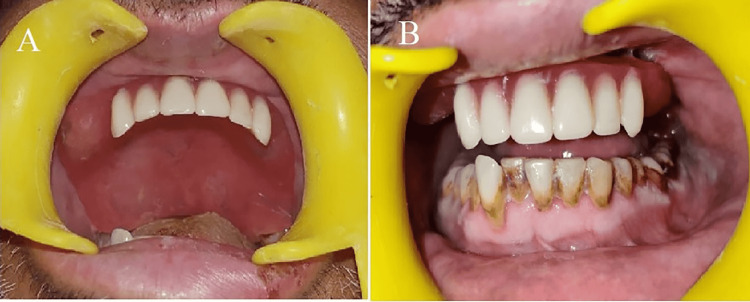
Prosthesis in situ (A) occlusal view (B) frontal view

The magnets were oriented on the working models (Figure 5). Then, using condensation silicon putty impression material (Zetaplus, Zhermack SpA, Badia Polesine (RO), Italy), the jaw relationship was recorded at an established occlusal vertical dimension (Figure 6). Next, the obturator was fabricated on the mounted models and processed using compression molding. During processing, the magnets in the obturator were embedded in space (a well) corresponding to the magnets’ location on the reconstruction plates (Figure 7). The processed obturator (Figure 8) had excellent retention, stability, and optimum spatial orientation because of the “novel technique.”

## Discussion

The treatment of mucormycosis in the orofacial region requires a combination of surgical debridement and antifungal therapy. Surgical debridement and resection of necrotic tissue can involve the palate, maxilla, nasal cartilage, and orbit [1,5]. This results in difficulty in speech, deglutition, mastication, and aesthetic concerns. The prosthetic rehabilitation of patients with bilateral maxillary resection presents a challenging situation due to the lack of availability of supporting bone and tissues. Moreover, the extent of the disease and multiple surgical debridements make the rehabilitation very complex and unpredictable. When defects are extensive, a multidisciplinary approach is needed to restore the palate, maxillae, and contiguous structures and restore function [6].

Osseointegrated implants have been successfully used to provide excellent retention, support, and stability of obturators. However, the high recurrence rate of mucormycosis and the need for multiple debridements contraindicate immediate implant or bone graft placement [7]. Moreover, immediate implant placement in the active osteomyelitis region may jeopardize osseointegration and the prognosis of rehabilitation because soft-tissue infections may progress deeper into the bone, undermining the osseointegration process. This necessitates a second surgery for implant placement after complete remission. The cost of treatment is also essential because of the high rate of recurrence and multiple surgeries [8].

Over the years, magnets have been used to retain definitive obturators [9]. The same principle has been used here for the spatial orientation of the obturator in maxillectomy. The design provides excellent stability, retention, and handling even when used with anterior teeth replacement. Like the surgical obturator, adding teeth to the postsurgical obturator should be limited to satisfying aesthetic needs and facilitating speech production. During the healing process, the obturator should not be intended for occlusal function since this is likely to result in movement of the obturator in and out of the defect, causing irritation and abrasion of the tissues in contact with the obturator [10].

The case was followed for four months. The advantage of the removable prosthesis is the ease of retrievability and continuous monitoring for potential recurrence of infection. This novel technique can provide a practical and cost-effective rehabilitation for mucormycotic maxillectomy cases.

## Conclusions

Mucormycosis requires immediate medical and surgical intervention because of its aggressive nature. Because surgical management alone compromises oral functions, a multidisciplinary approach is needed at this time. Using “reconstruction plates and magnets,” this novel technique offers a desirable rehabilitation option.
